# Rising Incidence of Glioblastoma Multiforme in a Well-Defined Population

**DOI:** 10.7759/cureus.8195

**Published:** 2020-05-19

**Authors:** Neil Grech, Theresia Dalli, Sean Mizzi, Lara Meilak, Neville Calleja, Antoine Zrinzo

**Affiliations:** 1 Department of Internal Medicine, Mater Dei Hospital, Msida, MLT; 2 Department of Neurosurgery, Mater Dei Hospital, Msida, MLT; 3 Department of Health Information and Research, Directorate for Health Information & Research, Pieta, MLT; 4 Department of Public Health, Faculty of Medicine & Surgery, University of Malta, Msida, MLT

**Keywords:** glioblastoma multiforme, incidence, survival, epidemiology, well-defined population

## Abstract

Background and Objectives

The incidence of glioblastoma multiforme (GBM) ranges from 0.59 to 5 per 100,000 persons, and it is on the rise in many countries. The reason for this rise is multifactorial, and possible contributing factors include an aging population, overdiagnosis, ionizing radiation, air pollution and others. The aim of this study is to conduct an epidemiological study of GBM in a well-defined population over a 10-year period and determine its significance, while comparing results with international standards.

Materials and Methods

All histological diagnoses of GBM in Malta from 2008 to 2017 were identified. Poisson regression was used to determine significance in incidence variation. Log-rank tests were used to compare the survival distributions of each variable. Cox regression for survival analysis with the Breslow method for ties was then performed to consider the overall model.

Results

A total of 100 patients (61 males; mean age 60.29±10.09 years) were diagnosed with GBM over the period 2008 to 2017. There was a significant increase in incidence from 0.73 to 4.49 per 100,000 over the 10-year period (p≤0.001). The most common presenting complaint was limb paresis (29%). Approximately 65% of patients were treated with maximum safe resection (MSR). Using Cox regression analysis, younger age at presentation and treatment with MSR significantly improved survival (p=0.026 and p≤0.001, respectively). The median survival was 10 months.

Conclusions

An increasing incidence of GBM is becoming evident, while the median survival remains low. This troubling trend emphasizes the importance of further research into GBM etiology and treatment.

## Introduction

Glioblastoma multiforme (GBM) is the most commonly occurring malignant primary brain tumor, representing 77%-81% of all primary malignant tumors of the central nervous system (CNS) [1]. It is classified as a grade IV diffuse astrocytic and oligodendroglial tumor by the World Health Organization [2]. The mean age of primary GBM presentation is 62 years, and the median survival is approximately 14.6 months [1,3]. The poor prognosis associated with GBM is well documented, while survival rates remain disappointingly low despite medical and surgical advances.

International studies reveal an approximate annual incidence rate of 0.59 to 5 per 100,000 persons; however, there have been studies indicating a rise in incidence [4-8]. Miranda-Filho et al. in 2017 described increasing rates of CNS and brain cancers in countries in South America, Eastern Europe and Southern Europe, while decreasing rates were only reported in Japan [9]. Dobes et al. in 2011 have also noted an increasing incidence of GBM tumors in two of their multicentered Australian studies, with a particular increase in frontal and temporal lobe GBM tumors [10].

The reasons for this increase in incidence are yet to be determined, and only possible causal factors can be postulated. Contributing factors may include an increase in diagnosis consequent to increasing ease of access to neuroimaging, an aging population, ionizing radiation, radiofrequency electromagnetic fields (RF-EMF) and air pollution, among others [11-18].

Malta is a small Southern European country with a population of approximately 475,000 people. Mater Dei Hospital (MDH) is Malta’s largest and only acute general hospital, which opened officially in 2007. Having one major hospital for the whole Maltese population, with little native population drift, allows for epidemiological national studies among a well-defined population, such as this one. This study, as opposed to many others, describes the epidemiology of GBM in a well-defined population.

We aimed to conduct an epidemiological study on GBM of Malta, a well-defined population, and to determine the significance of incidence trends over a 10-year period and compare results with international standards. We also aimed to determine the characteristics of all patients diagnosed histologically with GBM tumors, as well as assess which variables have a significant effect on survival.

## Materials and methods

All histological diagnoses of GBM from January 2008 till December 2017 were collected from the histology database of MDH. Information on an additional five cases was obtained from the Treatment Abroad office at MDH. These latter patients were operated in the United Kingdom, although postoperative care, including chemotherapy and/or radiotherapy, was administered in Malta. 

Each case history was reviewed using local electronic databases and online medical files. The data collected included patient demographics, presenting complaint, investigations, type of procedure performed, tumor position, molecular genetic testing and date of death, amongst others.

The data were inputted into the general-purpose statistical software system, STATA v12 (StataCorp LP, College Station, TX, USA). A time-trend analysis was calculated using Poisson regression. The system used log-rank tests to compare the survival distributions of each variable. The variables included gender, age of diagnosis, maximum safe resection (MSR), geographical regions, O^6^-methylguanine-DNA methyltransferase (MGMT) methylation testing, MGMT methylation presence, isocitrate dehydrogenase (IDH) 1 testing, IDH2 testing, reoperation, year of biopsy and time from presentation until time of biopsy. There were no IDH1- or IDH2-positive patients, and therefore their effect on survival could not be compared to IDH1/IDH2-negative patients. From the variables found to be statistically significant using the log-rank tests (taken as a p value less than 0.05), Cox regression for survival analysis with the Breslow method for ties was then performed to consider the overall model. 

## Results

**Figure 1:**
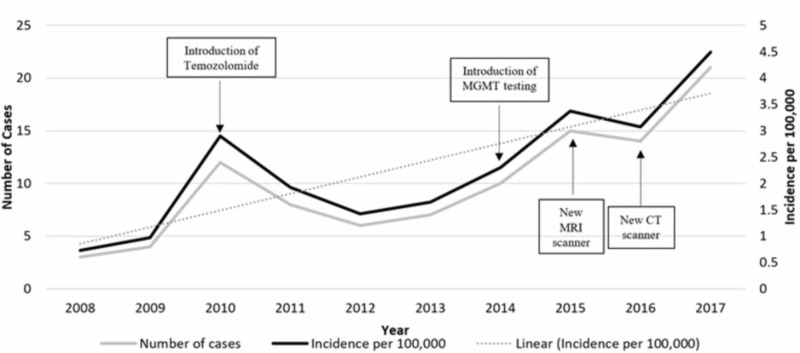
Cases and incidence of glioblastoma multiforme per year, with possible causal factors MGMT: O6-methylguanine-DNA methyltransferase

A total of 100 patients (61 males; 39 females) were diagnosed with GBM between 2008 and 2017 among the Maltese population. On averaging the incidence of GBM over 10 years, the incidence ratio of male to female is 1.39:089 per 100,000. From the five patients sent abroad for surgery, one patient was operated in 2009, three patients in 2010 and the final patient in 2011. A total of 21 patients were diagnosed with GBM in 2017 (incidence of 4.49 per 100,000), as opposed to only three patients diagnosed in 2008 (incidence of 0.73 per 100,000). There was a sudden increase in cases in 2010 and, however, a gradual increase from 2014 onward (Table 1; Figure 1). A time-trend analysis using Poisson regression revealed that the increase in incidence was significant with a p value of <0.001, with an incidence risk ratio of 1.16 per additional year (95% confidence interval [CI]=1.07-1.24).

**Table 1:** Cases and incidence of glioblastoma multiforme per year

Year	Cases	Incidence per 100,000 based on mid-year population
2008	3	0.73
2009	4	0.97
2010	12	2.90
2011	8	1.92
2012	6	1.43
2013	7	1.64
2014	10	2.30
2015	15	3.37
2016	14	3.07
2017	21	4.49

61% of the cohort were aged older than 60 years at presentation, with a mean age of 60.29±10.09 years. 29% (n=29) of patients presented with limb paresis (the most common presenting complaint), followed by headaches (n=19). Two patients (2%) were diagnosed incidentally on CT of the brain after presenting with mechanical falls and subsequent head injuries. The most common tumor position was the frontal lobe (25% [n=25]). 65% (n=65) of patients were treated with MSR (Table 2). MSR in our center refers to maximum resection of tumor with preservation of neurological function. Local techniques used to obtain MSR include intraoperative microscopy, and as from 2014, intraoperative neuronavigation was also introduced. All patients underwent a postoperative brain MRI within 48 hours to assess margins of resection.

**Table 2:** Characteristics of patients *Others including visual field defect, personality change, ataxia, syncope, sensory deficits and unknown presentation IDH: isocitrate dehydrogenase; MGMT: O^6^-methylguanine-DNA methyltransferase

Characteristic	
Gender, no. (%)	
Male	61 (61)
Female	39 (39)
Age at presentation, years	
Mean	60.29±10.09
Median	62
Range	28-80
Cases per 10-year age range, no. (%)	
20-29	1 (1)
30-39	3 (3)
40-49	10 (10)
50-59	25 (25)
60-69	47 (47)
70-79	13 (13)
80-89	1 (1)
Tumor position, no. (%)	
Frontal lobe	25 (25)
Parietal lobe	23 (23)
Temporal lobe	21 (21)
Multiple lobe involvement	21 (21)
Other (butterfly glioma, thalamus and brainstem)	6 (6)
Occipital lobe	4 (4)
Presenting complaint, no. (%)	
Limb paresis	29 (29)
Headaches	19 (19)
Seizures	9 (9)
Confusion	9 (9)
Cranial nerve palsy	7 (7)
Speech difficulty	6 (6)
Incidental finding	2 (2)
Other*	19 (19)
Maximum safe resection, no. (%)	
Yes	65 (65)
No	35 (35)
IDH1 status, no. (%)	
Present	0 (0)
Absent	15 (15)
Not tested	85 (85)
IDH2 status, no. (%)	
Present	0 (0)
Absent	12 (12)
Not tested	88 (88)
MGMT methylation, no. (%)	
Unmethylated (methylation rate for all the sites is less than 10%)	15 (15)
Low level of methylation (methylation rate for all the sites is 10% and 19%)	2 (2)
Methylated (methylation rate for all the sites is more than 20%)	5 (5)
Not tested	88 (88)

**Figure 2:**
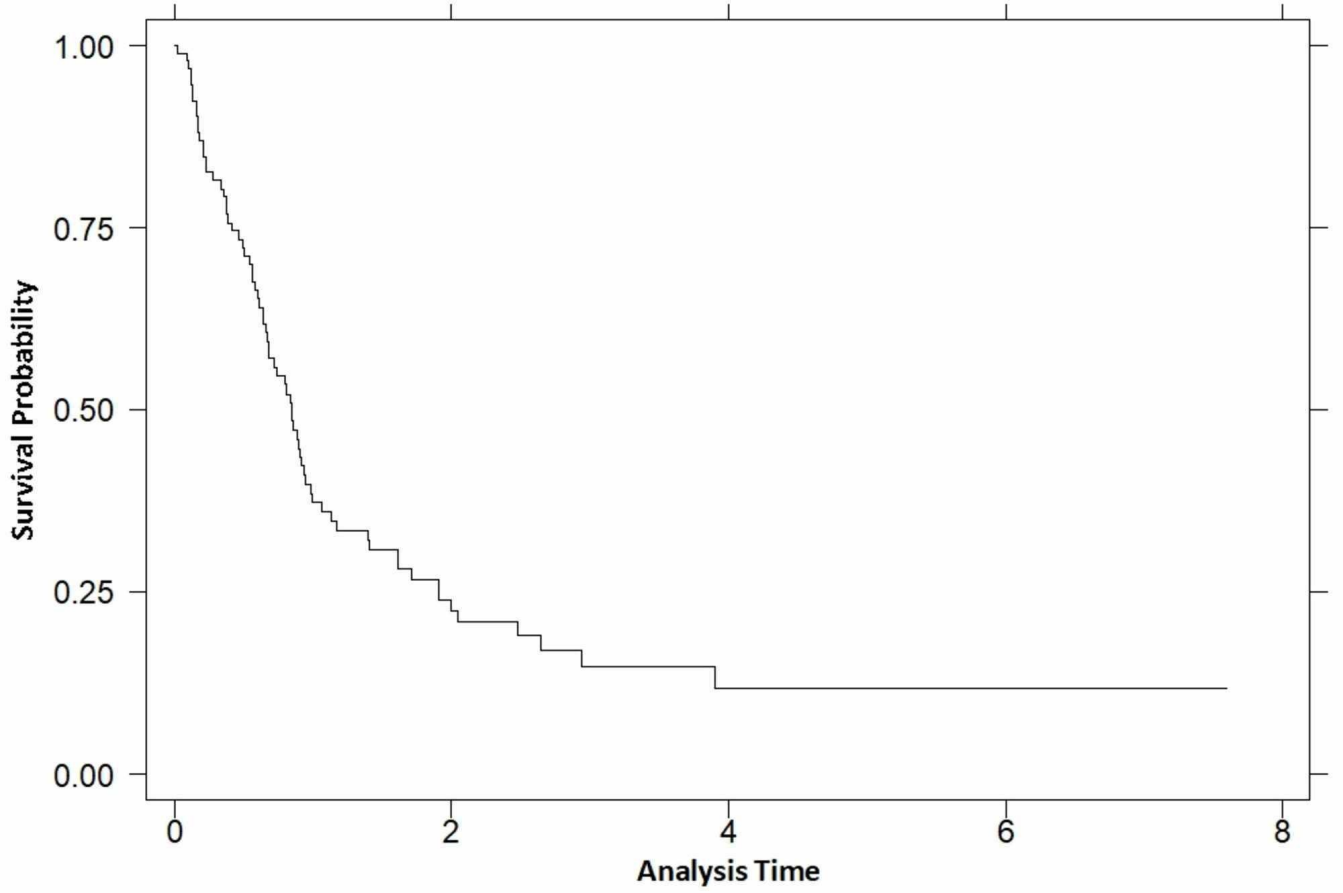
Kaplan-Meier curve of overall survival

A total of 78 patients passed away by the final date of data collection (3 March 2018) with a median survival of 10 months (Figure 2 for Kaplan-Meier curve of overall survival). Several variables were compared to determine their impact on survival (taken as a p value less than 0.05). From the variables mentioned above, two were found to have a significant effect on survival: age of diagnosis (p=0.019) and treatment with MSR (p≤0.001) (Table 3).

**Table 3:** Variables and their significance on survival *Although a significant value was calculated, the Kaplan-Meier curve showed overlap between the curves of the variable ‘geographical regions’; therefore, it could not be included in the multivariable cox regression. Together with the small sample sizes within each region, significance was lost MGMT: O^6^-methylguanine-DNA methyltransferase; IDH: isocitrate dehydrogenase

Variable	P value
Gender	0.199
Age of diagnosis	0.019
Geographical regions	<0.001*
Maximum safe resection vs no resection	<0.001
MGMT testing vs no testing	0.671
MGMT methylation status	0.074
IDH1 testing vs no testing	0.765
IDH2 testing vs no testing	0.857
Reoperation vs no reoperation	0.121
Year of biopsy	0.627
Time from presentation till time of tissue diagnosis	0.726

**Figure 3:**
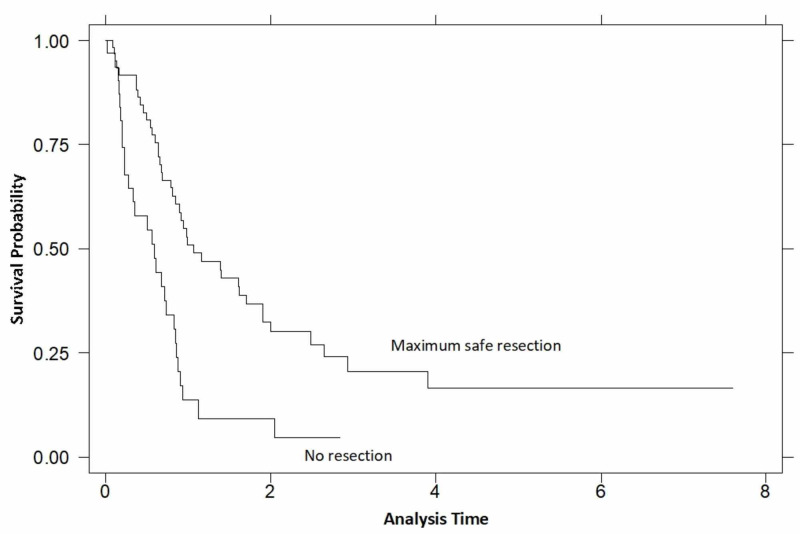
Kaplan-Meier survival estimates by resection

Using an overall model of Cox regression analysis, the two significant variables affecting survival (age of diagnosis and MSR) were tested for correlations. This further increased the statistical significance of these variables on their effect on survival, confirming they were independently correlated to survival (Table 4). The hazard ratio for the age of diagnosis was found to be 1.04, signifying an increased risk of death by 4% for every additional year the patient presented. Regarding MSR, if one were to undergo resection, survival time was found to be increased threefold (Figure 3).

**Table 4:** Overall model of Cox regression

Variable	Hazard ratio (95% confidence interval)	P value
Age of diagnosis	1.04 (1.013-1.067)	0.003
Maximum safe resection	3.03 (1.867-4.908)	<0.001

## Discussion

International literature cites the incidence of GBM ranging from 0.59 to 5 per 100,000 population, with rates increasing in various countries [4-10,19]. Reasons for such an increase may be multifactorial, and possible causal factors have been discussed in the literature. A possible contributing factor is related to an increase in neuroimaging over the past 20 years, apart from more accurate imaging techniques. Therefore, this has led to an overdiagnosis of brain tumors, as well as other tumor types [11]. Keeping GBM in mind, the most cited location is the frontal lobe, which often remains clinically silent. Hence, these patients tend to be discovered incidentally on imaging, therefore resulting in an increase of frontal lobe lesions [10,20].

Locally, two CT scanners and one MRI machine were in operation at MDH during 2008, with the addition of another MRI and CT scanner in 2015 and 2016, respectively (Figure 1). Approximately 14,000 CT scans were undertaken in 2008, whereas 38,000 scans were carried out in 2017. From the 14,000, more than half (7,641) were CT brains, and this increased to 11,466 CT brains in 2017. A significant increase in MRI scans was also noted, from 11,000 (1,315 MR head) in 2008 to 23,300 (4,757 MR head) scans in 2017.

Temozolomide chemotherapy agent was introduced in Malta in 2010. This agent is an alkylating chemotherapy and has become the first-line chemotherapy for histologically diagnosed GBM [3]. Naturally, its introduction led to a greater requirement for obtaining a histological diagnosis of brain tumors, and, in so doing, spuriously increasing the incidence rates of all types of brain tumors. This is also reflected by the fact that the majority of GBM cases diagnosed abroad were sent in 2010. Following this year, a second consultant neurosurgeon began working in 2011, along with the local introduction of advanced surgical equipment and software, therefore allowing for more complex surgeries to be performed locally, rather than being sent abroad. 

During 2014, for the first time in Malta, biopsies were sent abroad for MGMT testing. MGMT is a DNA repair enzyme, and its silencing by promoter methylation results in its decreased expression. This, in turn, results in increased responsiveness to the alkylating chemotherapy, temozolomide, and therefore, a longer survival following therapy [21]. Hence, this may have been a further incentive for obtaining a histological diagnosis (Figure 1).

Worldwide we are noting an increase in life expectancy, and along with this, an increase in various types of cancer. The life expectancy in Malta is rising and is averaged above 80 years of age, similar to other European countries [22]. Statistics showed that there has indeed been an increase from 2007 to 2017 of 57.8% of persons aged 65 years or older [23]. As previously discussed, the mean age of primary GBM presentation is 62 years, making this a concern with an increasingly aging population [1]. Trylcova et al. in 2015 discussed how aging processes may encourage a stable environment for glioblastoma tumor stroma and stimulate the invasive nature of this tumor in in vitro testing, via cancer-associated fibroblasts [13]. These findings were also supported by Smetana et al. in 2016 [12].

The etiology of GBM is unknown with the only identifiable risk factor being exposure to ionizing radiation. Previous exposure to ionizing radiation from CT scans increases the risk for all type of cancers, although Mathews et al. in 2013 found that brain cancer had the highest incidence rate ratio among all other cancers [15]. Preston et al. in 2007 found that the risk of radiation-associated cancer persists and is not dependent on age of exposure. They also concluded that glioma tumors particularly show a significant dose response [14]. As already mentioned, there has been an increase in CT brain scans performed in MDH over the years.

Another possible risk factor is RF-EMF, a type of non-ionizing radiation. Natural sources include the sun and the Earth itself, while artificial sources include satellite devices, broadcasting devices and mobile phones. A Working Group met in 2011 at the International Agency for Research on Cancer to assess the carcinogenicity of RF-EMF. They concluded that there may be an association between RF-EMF (level 2B evidence) and glioma predisposition [16]. Hardell and Calberg in 2015 found an association between mobile phone use and glioma risk with an odds ratio of 1.3 (95% CI=1.1-1.6 overall) to 3.0 (95% CI=1.7-5.2) [17]. According to the Malta Communications Authority reports, 385,636 active mobile phone internet subscriptions in 2008 had increased drastically to 604,759 subscriptions in 2017 [24]. There has also been an introduction of 4G in Malta in 2013, with coverage of the entire Maltese islands by 2015 [25]. Taking all this into consideration, there are clear indications of increasing public RF-EMF radiation levels throughout Malta. Currently, national risk assessments are being carried out to assess the introduction of 5G in Malta and other countries [26].

There is emerging evidence that another possible contributing factor in GBM etiology is air pollution. A large meta-analysis study including 12 European countries found suggestive evidence of a correlation between particulate matter ≤2.5 absorbance and malignant brain tumors. Having said this, among their limitations the authors admitted that they lacked certain information on brain cancer histology and could not directly assess the effects of pollution on glioblastoma type tumors against other non-malignant tumors [18]. The National Statistics Office of Malta has registered an average of 33 new vehicles per day. The total carbon dioxide equivalent emissions in Malta increased by 11.9% in 2017, when compared to 2010 levels [27]. Data published recently by Lelieveld et al. in 2019 have revealed that the poor air quality in Malta has led to 576 premature deaths in the year 2015, which averages at 137 early deaths per 100,000 (higher than the average European rate) [28].

Apart from discussing incidence trends and GBM’s possible etiology, other factors must also be considered and compared to the Maltese cohort. In the United States (US), there is a male predisposition to GBM with a male: female incidence ratio of 3.97:2.53 per 100,000. Therefore, males in the US have a 1.57 increased chance of suffering from GBM [1]. The most common location for GBM tumors in the US is the frontal lobe, followed by tumors overlapping multiple lobes and other supratentorial lobes [29]. The median survival remains low despite advances in medicine and is estimated at 14.6 months [3]. Prognostic factors that contribute to improved survival in GBM are methylation of the MGMT enzyme, younger age at the onset of disease, high Karnofsky Performance Status, high Mini-Mental State Examination score and increasing extent of resection boundaries [21,30]. Many similarities can be made between the above-mentioned findings and the findings in our study. 

A strength of our study is the fact that Malta has one major public hospital which accommodates all local neurosurgical operations, and therefore this allowed us to obtain a complete case capture of all GBM cases in our well-defined population. The study has a high specificity for GBM as we relied on histological diagnoses of GBM. Statistical significance has been calculated for the increase in incidence and other results.

The limitations of our study include a small cohort reflecting the size of the index population. No patients tested positive for IDH, and therefore its effect on survival could not be interpreted. We did not collect data regarding which patients received chemotherapy and radiotherapy, and therefore could not determine their effect on survival.

## Conclusions

The median survival of GBM remains low and despite medical advances, it continues to contribute significantly to mortality among patients diagnosed with CNS tumors. A worrying trend of increasing incidence of GBM is becoming evident although direct causes remain undetermined. This rising incidence emphasizes the importance of further research into the etiology and treatment of GBM tumors.
